# Ethical challenges and moral distress among field epidemiologists

**DOI:** 10.1186/s12889-022-12950-2

**Published:** 2022-03-16

**Authors:** Emma Cooke, George Lopez, Angela Hilmers, David G. Addiss

**Affiliations:** 1 School of Medicine and Ethics Center, Emory University, Atlanta, GA 30322 USA; 2 Rollins School of Public Health, Emory University, Atlanta, GA 30322 USA; 3 TEPHINET, The Task Force for Global Health, 325 Swanton Way, Decatur, GA 30030 USA; 4 Focus Area for Compassion and Ethics, The Task Force for Global Health, 330 W. Ponce de Leon Ave, Decatur, GA 30030 USA

**Keywords:** Ethics, Field epidemiology, Global health, Moral distress, Public health

## Abstract

**Background:**

As ‘disease detectives’ and directors of public health programs, field epidemiologists play essential roles in protecting public health. Although ethical issues receive considerable attention in medical and research settings, less is known about ethical challenges faced by field epidemiologists in public health programs. Similarly, little is known about moral distress among field epidemiologists, i.e., situations in which they are constrained from acting on what they know to be morally right. Moral distress is strongly associated with empathy fatigue, burnout, reduced job retention, and disengagement. To better understand ethics training needs for field epidemiologists, in February 2019, members of TEPHIConnect, an online and mobile networking platform for Field Epidemiology Training Program (FETP) alumni, were invited to participate in an anonymous survey about ethical challenges and moral distress.

**Results:**

Among 126 respondents from 54 countries, leading causes of ethical dilemmas included inadequate informed consent (61%), inequitable allocation of resources (49%), and conflicts of interest (43%). These occur primarily in settings of disease outbreaks (60%); research (55%); and public health programs at the state, province, or national level (45%) or community level (43%). Work-related moral distress was reported by 91% of respondents, including 26% who experience it “frequently” or “almost always.” Field epidemiologists working in low- and low-middle income countries were more likely to report moral distress “frequently” or “almost always” than those in higher-income countries (33.0% vs 9.1%, *P* = 0.006). The most common perceived contributors to moral distress included excessive stress and work demands (30%) and inadequate support from leaders (25%).

**Conclusions:**

Field epidemiologists face significant work-related ethical challenges, which are endemic to public health and political systems. A substantial proportion of field epidemiologists also experience some degree of moral distress, often in association with these challenges. These findings indicate an unmet need among field epidemiologists for support in navigating ethical challenges, as well as for resources to address the human and professional consequences of moral distress.

**Supplementary Information:**

The online version contains supplementary material available at 10.1186/s12889-022-12950-2.

## Introduction

Public health programs rely on field epidemiologists for program design, implementation, monitoring, and evaluation. Field epidemiologists are employed by state and national health departments to identify, prevent, and respond to public health threats [1]. Occupying dual roles as ‘disease detectives’ and administrators of routine public health programs, field epidemiologists face ethical challenges that have not been well-studied. The many interests, influences, and pressures in public health render these ethical challenges particularly complex.

Public health workers often encounter health threats that seem intractable, particularly those rooted in the social determinants of health [2]. As such, they are subject to moral distress. Moral distress was initially defined by Jameton as “the psychological distress of being in a situation in which one is constrained from acting on what one knows to be right” [3]. Moral distress has been characterized primarily in nurses and other healthcare workers, as well as in first responders and humanitarian settings [4–8]. Moral distress is associated with all three dimensions of burnout (emotional exhaustion, depersonalization, and decreased personal accomplishment) [5], and has been linked to poor work performance and staff turnover [6].

Despite the robust literature on moral distress in clinical and direct-care settings, relatively little is known about moral distress in public health field work. The few studies that exist have focused largely on community health workers involved in HIV care or health workers in the 2014–2016 Ebola epidemic [2, 7]. In these settings, moral distress was linked to “an inability to meet an overwhelming demand for basic patient care needs with limited supplies and other resources,” as well as conflict between maintaining personal safety and providing care for others [7]. Community health and health promotion workers in non-clinical settings have expressed feelings of moral distress related to their inability to change upstream governmental policies that they felt were at the root of negative health outcomes [2, 9]. A recent study reported high burdens of occupational stress among field epidemiology trainees [10], and public health officials have been subjected to harassment and threats of violence during the COVID-19 pandemic [11, 12]. However, to our knowledge, moral distress has not been well-documented or quantified among practicing field epidemiologists, who, because of their central role and position within public health systems, have a unique vantage point on ethical challenges and dilemmas in public health.

## Methods

To better understand the ethical challenges faced by field epidemiologists and to guide the potential development of curricula for ethics training, we asked practicing field epidemiologists to complete a survey. Seventy-three field epidemiology training programs (FETPs) currently provide epidemiologic training for ministries of health in at least 100 countries. Patterned on the Epidemic Intelligence Service training at the US Centers for Disease Control and Prevention with an emphasis on practical, on-the-job training [1], FETPs are linked in a global network, TEPHINET, with a secretariat at the Task Force for Global Health. Since 2017, FETP alumni have been connected through an online networking platform, TEPHIConnect, which in February 2019 had 1368 subscribers. On February 25, 2019 an invitation to participate in a survey related to ethical challenges was posted on TEPHIConnect, along with a link to a survey that used Qualtrics software. The link was available to respondents for one month; no reminder notices were issued.

This anonymous 13-question survey requested information on position and setting of employment and year of FETP training. Respondents were given a fixed menu of options and asked to select up to three “ethical challenges” that they encounter (Supplemental appendix), as well as the settings in which they experience them. Respondents also were asked to indicate three such challenges for which they would most value training and opportunities to build skills and knowledge, as well as the preferred format for such training. Using the definition of moral distress as a situation in the workplace in which “you know the morally correct thing to do, but circumstances or competing claims prevent you from doing it,” respondents were asked using a Likert scale how frequently they experienced moral distress in their work. Those who reported experiencing moral distress in the workplace were invited to describe the situations most frequently associated with moral distress. The narrative responses to this open-ended question were reviewed for common themes.

Data were analyzed using SAS software (SAS Institute Inc. 2013). The ***χ***^**2**^ test was used to assess statistical significance.

## Results

A total of 126 field epidemiologists from 54 countries completed the survey. Of these, 49 (39%) were physicians and 108 (86%) worked primarily within public health programs (Table 1). Of the 126 respondents, 102 (81%) engaged in epidemiologic surveillance, 53 (42%) participated in training, and 49 (39%) participated in research. Thirty-seven (29%) respondents live and work in low-income countries, 54 (43%) in low-middle-income countries, and 35 (28%) in high-income countries.

**Table 1:** Characteristics of Field Epidemiology Training Program (FETP) alumni who participated in the survey (*n* = 126)

Characteristic	*n* (%)
**Country income category **^**a**^	
Low	37 (29)
Lower-middle	54 (43)
Upper-middle and High	35 (28)
**Year of FETP graduation **^**b**^	
2014 and before	41 (34)
2015–2017	45 (37)
2018 and after	35 (29)
**Highest academic degree**	
MPH or equivalent	49 (39)
Clinical (e.g., MD, RN, PA)	55 (44)
Non-clinical (e.g., PhD, MS)	22 (17)
**Professional setting**	
Health program	108 (86)
Research or academic	18 (14)
**Work-related activities **^**c**^	
Administration	33 (26)
Research	49 (39)
Training	53 (42)
Health surveillance	102 (81)
Clinical care	14 (11)
Other	14 (11)

^a^As defined by The World Bank. Countries considered as upper-middle and high-income countries by the World Bank are listed here as high-income

^b^Missing 5 responses

^c^Participants could select up to 3 activities

### Ethical challenges

The most commonly encountered work-related ethical issues included lack of informed consent (77 respondents, 61%), unjust allocation of resources (62 respondents, 49%), and conflicts of interest (54 respondents, 43%) (Table 2). Respondents were asked to select up to three settings in which they most commonly encounter ethical issues or dilemmas. These settings included outbreaks of infectious disease (75 respondents, 60%), research studies (69, or 55%), and public health programs organized at the state, province, or national level (57, or 45%) (Table 3). Neither the specific types of ethical issues nor the settings in which they occur were significantly associated with gender, year of graduation, academic degree, work type (health program vs. research), or work engagement activity.

**Table 2:** Primary ethical dilemmas encountered at work among 126 field epidemiologists ^a^

Category	*n* (%)
Inadequate informed consent	77 (61)
Inequitable allocation of health resources	62 (49)
Conflicts of interest	54 (43)
Corruption	36 (29)
Lack of community engagement in public health measures	35 (28)
Inadequate access to health care	30 (24)
Occurrence of unintended harm	11 (9)

^a^Participants selected up to 3 responses; two responses missing

**Table 3:** Settings in which 126 field epidemiologists encountered ethical dilemmas^a^

Category	*n* (%)
Infectious disease outbreak	75 (60)
Research	69 (55)
State, province, or national-level public health program	57 (45)
Community-level public health program	52 (41)
Clinical care	30 (24)

^a^Participants could select up to 3 settings, missing 1 response

Among 126 respondents, 64 (51%) expressed a desire for further training on the ethics of informed consent and 66 (52%) requested training on addressing inequity. Thirty-three (26%) respondents desired training on increasing community autonomy and 32 (25%) expressed a desire for training on how to address corruption. Among four potential formats for ethics training, the most highly preferred option was webinars (35%), followed by inclusion of ethics training within FETP curricula (21%), online e-learning courses that provide certificates upon completion (20%), and in-person workshops at FETP conferences (20%).

### Moral distress

Thirty-three (26%) respondents reported that they experience moral distress in the workplace “almost all the time” (5%) or “frequently” (21%), and 82 (65%) reported experiencing moral distress “sometimes.” Eleven (9%) respondents reported not experiencing moral distress at work. Among 91 respondents from low and lower-middle income countries, 30 (33%) reported experiencing moral distress frequently or all the time, compared to three (9.1%) from higher-income countries (*p* = 0.006).

Eighty subjects responded to an open-ended question asking them to describe specific situations that lead to moral distress in the workplace. The most common responses reflected three major themes: excessive work-related demands and stress (30%); supervisors or leaders who are unsupportive, ineffective, or corrupt (25%); and inadequate resources or poor access to public health services (23%). Examples of responses for each theme are shown below.

#### Excessive demands and stress

*“Excessive work load; pressure from higher-ups.”*

*“When working under pressure just trying to meet deadlines.”*

#### Unsupportive, ineffective, or corrupt leaders

*“Interference from political and other leaders…”*

*This happens mostly in situations where something that is beneficial for public health is being planned and a non-technical or corrupt head of the department discourages the intervention and imposes his or her non-beneficial interventions.”*

*“When senior colleagues ask you to stay silent over an issue and you let wrong decisions be taken just to protect some specific person or interest.”*

*“Most of the time, higher administrators do not understand the usefulness of preventive approaches… they think it is a waste of resources and ignore steps until things become emergencies.”*

#### Inadequate resources and poor access to services

*“It is demoralizing to be in a situation in which one is eager to carry out his or her moral and professional responsibilities with minimal infrastructure to do so.”*

*“Since I am currently at the university I spend most of my time teaching. However, as I conduct research, I come across situations in which the subjects may not be accessing the health facilities they need. More often than not, I will not be in a position to rectify the situation or even make any promises.”*

*“During field outbreak responses, everything unrelated to that outbreak falls by the wayside, and often someone who doesn’t have the specific disease in question doesn’t get the care they need.”*

Respondents also described participating in actions that violated their ethical principles and being restrained from acting in ways that reflected their values.

*“During vaccination campaigns, when a parent refuses to have his child vaccinated, engaging in procedures to ensure that the child is vaccinated.”*

*“When I know that it should be done but I am not empowered to do it”*

*“Inability to be transparent with data…”*

## Discussion

Field epidemiologists occupy a crucial niche within public health. As civil servants, they are generally one step removed from the suffering of individual patients and are involved in improving population health through evidenced-based programs and policies [1]. These efforts can be thwarted by lack of attention, partisan interests, or corruption among government leaders and within the agencies where epidemiologists work [12]. Thus, the same public institutions that authorize public health interventions and create the conditions for remarkable achievements can, at times, threaten public health and well-being. The sometimes uneasy relationship between field epidemiologists, committed to evidence-based action, and the politically-derived authority of the institutions in which they serve gives rise to a range of ethical dilemmas. If unaddressed, these ethical challenges can lead to moral distress and burnout, and to the failure of public health systems.

### Ethical challenges

To effectively respond to the moral challenges faced by field epidemiologists, it is necessary to clarify the specific circumstances where ethical dilemmas and moral distress arise, as well as the underlying value conflicts that give rise to them. In our survey, field epidemiologists identified inadequate informed consent, inequitable allocation of health resources, and conflicts of interest as primary ethical challenges.

These ethical challenges were most commonly reported in research settings and infectious disease outbreaks. Ethical lapses in biomedical research during the last century led to safeguards intended to protect human research subjects, such as ethics review committees and institutional review boards [13, 14]. These safeguards have been important in medicine and public health, and are still evolving, although critics have argued that they leave key elements unaddressed, particularly with regard to community engagement and issues of fairness [15, 16]. As the results of our survey show, field epidemiologists are aware of these gaps, not only within the context of community-based implementation research, but more acutely, within public heath practice. For example, the emphasis on informed consent in the context of human subjects research does not always extend to public health practice, particularly for population-level interventions [17].

Currently, ethics training in FETP curricula focuses on scientific integrity, ethical principles, and the role of IRBs in human subjects research (A. Hilmers, personal communication). These are important topics, and they are covered in many university-level public health ethics courses. However, survey respondents expressed a need for additional guidance and practical skills for navigating the fundamental structural barriers and moral ambiguities that arise in the day-to-day work of public health, particularly related to health inequities, limited access to care, community autonomy, conflicts of interest, and corruption.

Previous work has highlighted the pressing ethical challenges of outbreak investigations, where a timely response is essential, circumstances are rapidly evolving, multiple stakeholders (often not fully identified) have competing interests, and the stakes are high, both for human health and for the reputations of epidemiologists and their institutions [18, 19]. The World Health Organization has developed ethical guidelines for infectious disease outbreak investigations, including recommendations for allocating resources, balancing public health with individual privacy and civil liberties, and utilizing experimental treatments, among other topics [18]. International working groups have also highlighted regional differences in response to pandemics, as well as the importance of transparency and inclusiveness in responding to outbreaks rather than a mandated, ‘one-size-fits-all’ approach [19]. These resources provide a helpful framework for field epidemiologists, but our results suggest that further guidance and training are needed. The same challenges that make outbreaks difficult to respond to also make it difficult to develop specific advance guidelines. For this reason, proposals have been made to integrate ethical analysis into public health practice [20]. Incorporating practical ethics into field epidemiology training would give field epidemiologists the background knowledge and ethical foundation needed to respond to rapidly developing outbreaks. This training would also fulfill survey respondents’ expressed desire for further ethics education.

### Moral distress

Our findings indicate that a high proportion of field epidemiologists, 91%, experience work-related moral distress, with 5% reporting moral distress “almost all the time” and 21% reporting it “frequently.” Respondents from low and low-middle income countries were more than three times more likely to report frequent moral distress than those from higher-income countries. It is not clear whether this difference is associated with differences in moral sensitivity, institutional safeguards, structural barriers to ethical responsiveness, or other factors.

The most commonly reported sources of moral distress included structural factors such as excessive work-related demands and stress, unsupportive or ineffective leadership, and lack of resources. Similar factors (on the national, state, and institutional levels) have been cited as sources of moral distress among frontline COVID-19 workers [21]. Thus, far from being a theoretical concern, moral distress in field epidemiologists appears to be a direct response to the daily circumstances encountered in the workplace.

Respondents to the survey identified infectious disease outbreaks as the most frequent setting of ethical dilemmas. This finding is pertinent in light of the COVID-19 pandemic, which began almost a year after the survey was conducted. Up to 72 percent of COVID-19 frontline health workers in Wuhan, China have reported emotional distress [22], while half of all Italian health workers reported symptoms of post-traumatic stress related to caring for COVID patients [23]. Moral distress likely plays a role in these emotional difficulties, as healthcare workers must make ‘no-win’ decisions (e.g. choosing which patients will be eligible for a ventilator), or are forced to take on responsibilities outside their normal scope of practice [24]. These challenges can also be seen in other recent outbreaks: healthcare workers in West Africa’s 2014–2016 Ebola outbreak similarly struggled with allocating scarce resources and balancing personal risk with a sense of obligation to patients [7]. Growing awareness of moral distress, particularly in the setting of COVID-19, has led some health systems to implement initiatives such as peer counseling and “oasis rooms” where health workers can rest and recharge [25]. During outbreaks, field epidemiologists are often recruited to the frontline response in various capacities; they may benefit from similar initiatives.

Moral distress can lead to burnout, decreased self-esteem, and depression [5], and can prompt healthcare workers to leave or consider leaving their jobs [6, 12]. Highly-charged scenarios such as infectious disease outbreaks lead to moral distress, but the daily experience of public health field work can do so as well, particularly since people may enter the field with idealism that clashes with the reality they find on the ground [9].

This geographically diverse survey adds field epidemiologists to the growing list of professions vulnerable to moral distress. Our survey was limited by a relatively small sample size, a low response rate, a non-random sample, and lack of information on non-respondents. In the interests of brevity, we did not collect potentially useful information, such as respondents’ ethics training outside the FETP. Although the findings cannot be generalized to all practicing field epidemiologists, they point towards the importance of recognizing moral distress in field epidemiologists and the need for training that empowers them to respond to complex ethical challenges.

### Conclusions

Field epidemiologists face significant work-related ethical challenges, which are endemic to public health and political systems. A substantial proportion of field epidemiologists experience some degree of moral distress, often in association with these challenges. These findings indicate an unmet need among field epidemiologists for support in navigating ethical challenges, as well as for resources to address the human and professional consequences of moral distress.

## Supplementary Information

**Additional file 1. **

## Data Availability

Survey data are available from the corresponding author**.**

## References

[1] Goodman RA, Buehler JW, Mott JA, Rasmussen SA, Goodman RA (2019). Defining field epidemiology. The CDC field epidemiology manual.

[2] Maes K, Kalofonos I (2013). Becoming and remaining community health workers: Perspectives from Ethiopia and Mozambique. Soc Sci Med.

[3] Jameton A (2017). What moral distress in nursing history could suggest about the future of health care. AMA J Ethics.

[4] Epstein EG, Hamric AB (2009). Moral distress, moral residue, and the crescendo effect. J Clin Ethics.

[5] Rushton CH, Batcheller J, Schroeder K, Donohue P (2015). Burnout and resilience among nurses practicing in high-intensity settings. Am J Crit Care.

[6] Austin CL, Saylor R, Finley PJ (2016). Moral distress in physicians and nurses: impact on professional quality of life and turnover. Psychol Trauma Theory Res Pract Policy.

[7] Ulrich CM. Ebola is causing moral distress among African healthcare workers. BMJ 2014:349 10.1136/bmj.g6672 25380700

[8] Gustavsson ME, Arnberg FK, Juth N, von Schreeb J (2020). Moral distress among disaster responders: what is it?. Prehosp Disaster Med.

[9] Sunderland N, Harris P, Johnstone K, Fabbro LD, Kendall E (2014). Exploring health promotion practitioners’ experiences of moral distress in Canada and Australia. Glob Health Promot.

[10] Ryu S, Kim YW, Kim S, Liao Q, Cowling BJ, Lee C-S (2019). Occupational stress among field epidemiologists in Field Epidemiology Training Programs from the Public Health Sector. Int J Environ Res Public Health.

[11] Mello MM, Greene JA, Sharfstein JM (2020). Attacks on public health officials during COVID-19. JAMA.

[12] Smith MR, Weber L. Health officials are quitting or getting fired amid outbreak. AP News, August 10, 2020. https://apnews.com/article/virus-outbreak-u-s-news-ap-top-news-ok-state-wire-ca-state-wire-8ea3b3669bccf8a637b81f8261f1cd78. Accessed May 6, 2021.

[13] Rodriguez MA, Garcia R (2013). First, do no harm: the US sexually transmitted disease experiments in Guatemala. Am J Public Health.

[14] Moon MR (2009). The history and role of institutional review boards: a useful tension. Virtual Mentor.

[15] Lavery JV, Ijsselmuiden C (2018). The Research Fairness Initative: Filling a critical gap in global research ethics. Gates Open Research.

[16] Tindana PO, Singh JA, Tracy CS, Upshur REG, Daar AS, Singer PA (2007). Grand challenges in global health community engagement in research in developing countries. PLoS Medicine.

[17] Addiss DG, Kienast Y, Lavery JV (2021). Ethical dimensions of neglected tropical disease programming. Trans Roy Soc Trop Med Hyg.

[18] World Health Organization (2016). Guidance for managing ethical issues in infectious disease outbreaks.

[19] Lor A, Thomas JC, Ortmann DHBW, Guibert DJH (2016). Key ethical issues discussed at CDC-sponsored international, regional meetings to explore cultural perspectives and contexts on pandemic influenza preparedness and response. Int J Health Policy Manag.

[20] Levin BW, Fleischman AR (2002). Public health and bioethics: the benefits of collaboration. Am J Public Health.

[21] Cacchione PZ (2020). Moral distress in the midst of the COVID-19 pandemic. Clin Nurs Res.

[22] Lai J, Ma S, Wang Y, Cai Z, Hu J, Wei N (2020). Factors associated with mental health outcomes among health care workers exposed to coronavirus disease 2019. JAMA Network Open.

[23] Rossi R, Socci V, Pacitti F, Di Lorenzo G, Di Marco A, Siracusano A (2020). Mental health outcomes among front and second line health workers associated with the COVID-19 pandemic in Italy. Jama Network Open.

[24] Greenberg N, Docherty M, Gnanapragasam S, Wessely S (2020). Managing mental health challenges faced by healthcare workers during covid-19 pandemic. BMJ.

[25] Hoffman J (2020). ‘I can’t turn my brain off’: PTSD and burnout threaten medical workers. NY Times.

