# Supplementary material for: Ethical challenges and moral distress among field epidemiologists
Source: BMC Public Health. 2022 Mar 16;22:510. doi: 10.1186/s12889-022-12950-2 (PMC8924561; doi:10.1186/s12889-022-12950-2)
Supplement: Supplementary file 1 — Additional file 1. [file 12889_2022_12950_MOESM1_ESM.pdf]

Supplemental appendix. Survey completed by 126 field epidemiologists, 2019.

Section 1: Training and current work

The purpose of this survey is to ask questions about ethical challenges that FETP alumni experience in their work. The data that you and other alumni provide will help to develop Ethics learning courses and resources for the FETP community. Your participation is voluntary and your responses are completely confidential. Additionally, your responses are combined with those of many others and summarized in a report to further ensure confidentiality. All data from this survey will be stored in a secure server with access provided only to key personnel.

1) In what country do you currently live?

2) In which FETP or applied epidemiology training program did you receive your training?

3) What is your year of graduation or expected graduation?

4) Which of the following degree(s) do you hold?
   a) MD
   b) RN
   c) PhD
   d) DVM
   e) MPH
   f) Other (please list)

5) What is your current professional role or position?

6) In what type of work are you currently engaged? Please check all that apply.
   a) Administration
   b) Research
   c) Training
   d) Public health/Health surveillance
   e) Clinical care
   f) Other

Section 2: Ethical issues

This section asks questions about issues and challenges that you encounter in your work that have ethical implications. This would include issues that you perceive to contain a moral dimension, that challenge or upset your established values, or that engage questions of what is right, wrong, and realistically possible in a given setting.

7) In which of the following settings do you most frequently encounter ethical issues or dilemmas? Please select a maximum of three.
   a) Patient care
   b) Research
   c) National or district-level public health programs
   d) Community-level interventions
   e) Outbreak investigations
   f) Other (please describe)

8) Which ethics-related issues do you most commonly encounter in these settings? Please select a maximum of three.
   a) Informed consent *(e.g. situations where patients are inadequately told about risks, benefits, and alternatives to treatment)*
   b) Inadequate access to essential drugs and health services *(e.g. situations where patients cannot get medications or treatments they need)*
   c) Inequitable allocation of public health resources *(e.g. resources being distributed unfairly; treatments or resources not going to those who need them most)*
   d) Individuals or animals being harmed by public health interventions *(e.g. side effects or undesirable outcomes from mass drug administration or other public health interventions)*
   e) Lack of autonomy among community members or “beneficiaries” of health interventions *(e.g. community members not getting to say no to treatment, or having few choices about treatments)*
   f) Corruption *(e.g. people in power behaving in a dishonest or unscrupulous way; threats; bribery)*
   g) Conflicts of interest *(e.g. people's financial interests pushing them away from "doing the right thing" or acting in the best interest of patients and/or community members)*
   h) Other (please describe)

9) For which of the issues listed in the previous question would you value training or opportunities for building your skills or knowledge? Please select a maximum of three.

a) Informed consent *(e.g. situations where patients are inadequately told about risks, benefits, and alternatives to treatment)*

b) Inadequate access to essential drugs and health services *(e.g. situations where patients cannot get medications or treatments they need)*

c) Inequitable allocation of public health resources *(e.g. resources being distributed unfairly; treatments or resources not going to those who need them most)*

d) Individuals or animals being harmed by public health interventions *(e.g. side effects or undesirable outcomes from mass drug administration or other public health interventions)*

e) Lack of autonomy among community members or "beneficiaries" of health interventions *(e.g. community members not getting to say no to treatment, or having few choices about treatments)*

f) Corruption *(e.g. people in power behaving in a dishonest or unscrupulous way; threats; bribery)*

g) Conflicts of interest *(e.g. people's financial interests pushing them away from "doing the right thing" or acting in the best interest of patients and/or community members)*

h) Other (please describe)

10) In what format would you prefer to receive this training? Rank the following options in order of preference, 1 being your most preferred format to receive training.

______ Webinar
______ Online e-learning course that provides certificate upon completion
______ In-person workshop at FETP conference
______ Part of FETP training curriculum

11) In the workplace, to what extent do you find yourself in situations of moral distress (where you know what the morally correct thing is to do but circumstances or competing claims prevent you from doing it)?

Never

Sometimes

Frequently

Almost all the time – I deal with this constantly

12) When you experience moral distress, what are the situation(s) that most frequently cause it? *[question automatically skipped if respondent answered "never" to previous question]*

13) What additional information regarding ethics would you find helpful to your work?

14) Optional - if you would like to discuss this survey further, please email face@taskforce.org.
